# Supplementary material for: Brain adiponectin signaling controls peripheral insulin response in Drosophila
Source: Nat Commun. 2021 Sep 24;12:5633. doi: 10.1038/s41467-021-25940-6 (PMC8463608; doi:10.1038/s41467-021-25940-6)
Supplement: Supplementary file 1 — Supplementary informations [file 41467_2021_25940_MOESM1_ESM.pdf]

Supplementary information

Genotypes corresponding to animals of the Figures and supplemental Figures

Fig. 1b–c:	w^-;+; Apn-Gal4/UAS-GFP-RNAi
	w^-;+; Apn-Gal4/UAS-AdipoR-Ri.
Fig. 1d:	w^-/Y;+; Apn-Gal4/UAS-GFP-RNAi
	w^-/Y;+; UAS-AdipoR-Ri/UAS-GFP-RNAi
	w^-/Y; UAS-AdipoR-act/+;UAS-GFP-RNAi/+
	w^-/Y;+; Apn-Gal4/UAS-AdipoR-Ri
	w^-/Y; UAS-AdipoR-act/+; Apn-Gal4/+
	lpp-Gal4/Y; +; UAS-GFP-RNAi/+
	w^-/Y; UAS-hAdipoQ/+; UAS-GFP-RNAi/+
	lpp-Gal4/Y; UAS-hAdipoQ/+; +
Fig. 1e–g:	w^-; lexAop-CD8-GFP-2A-CD8-GFP/+;UAS-mLexA-VP16-NFAT, LexAop-rCD2-GFP/Apn-Gal4
Fig. 1f:	w^-; lexAop-CD8-GFP-2A-CD8-GFP/+;UAS-mLexA-VP16-NFAT, LexAop-rCD2-GFP/Apn-Gal4, UAS-AdipoR-Ri
Fig. 2a:	w^-;+; UAS-GFP-RNAi
Fig. 2b:	w^-;+; UAS-GFP-RNAi
Fig. 2d:	lpp-Gal4/+/Y; +; UAS-GFP-RNAi/+
	lpp-Gal4/+/Y; +; UAS-grp78-RNAi/+
Fig. 3a:	w^-; UAS-mCD8-GFP/+;Apn-Gal4/+
Fig. 3b–c:	w^-;+; Apn-Gal4/UAS-GFP-RNAi
	w^-;+; Apn-Gal4/UAS-AdipoR-Ri.
Fig. 3d–e:	w^-;+; UAS-GFP-RNAi
Fig. 3f–g:	met^{27}, gce^{25k}/+; +; Apn-Gal4/UAS-GFP-RNAi
	+/FM7; +; Apn-Gal4/UAS-GFP-RNAi
	met^{27}, gce^{25k}/+; +; Apn-Gal4/UAS-AdipoR-Ri
	+/FM7; +; Apn-Gal4/UAS-AdipoR-Ri
Fig. 4a–a':	w^-;tGPH/+; Apn-Gal4/UAS-GFP-RNAi
	w^-;tGPH/+; Apn-Gal4/UAS-AdipoR-Ri
Fig. 4b:	w^-;tGPH/+; UAS-GFP-RNAi/+
Fig. 4c:	elav-Gal4/+/Y; +; gd2HF/UAS-GFP-RNAi
	elav-Gal4/+/Y; +; gd2HF/UAS-AdipoR-Ri
	elav-Gal4/+/Y; UAS-AdipoR-act/+; gd2HF/+
	w^-; gd2HF/+; Apn-Gal4/UAS-GFP-RNAi
	w^-; gd2HF/+; Apn-Gal4/UAS-AdipoR-Ri
	w^-; gd2HF/+; Apn-Gal4/UAS-AdipoR-TRIP
	w^-; gd2HF/+; Apn-Gal4/UAS-AdipoR-act
Fig. 4d:	lpp-Gal4/+/Y; +; gd2HF/+
	lpp-Gal4/+/Y; UAS-hAdipoQ/+; gd2HF/+
Fig. 4e:	yw-; +; gd2HF/UAS-GFP-RNAi
Fig. 4f–g:	w^-;+; Apn-Gal4/UAS-GFP-RNAi
	w^-;+; Apn-Gal4/UAS-AdipoR-Ri

Fig. 4h–i:	lpp–Gal4/+/Y; +;gd2HF/+
	lpp–Gal4/+/Y; +;gd2HF/UAS–gfp78–Ri
	lpp–Gal4/+/Y; UAS–grp78/+;gd2HF/+
Fig. S1a:	w^-; UAS–mCD8–GFP/+;Apn–Gal4/+
Fig. S1b:	w^-;+; UAS–GFP–RNAi
Fig. S1c:	w^-;+; Apn–Gal4/UAS–GFP–RNAi
	w^-;+; Apn–Gal4/UAS–AdipoR–Ri
Fig. S1d:	w^-; ptc–Gal4/UAS–AdipoR–Myc; +
Fig. S1e	w^-;+; Apn–Gal4/UAS–nuclearGFP
Fig. S1f:	w^-;+; Apn–Gal4/UAS–GFP–RNAi
	w^-;+; UAS–AdipoR–Ri/UAS–GFP–RNAi
	w^-; UAS–AdipoR–TRIP/+;UAS–GFP–RNAi/+
	w^-;+; Apn–Gal4/UAS–AdipoR–Ri
	w^-; UAS–AdipoR–TRIP/+; Apn–Gal4/+
Fig. S1g:	w^-;+; Apn–Gal4/UAS–GFP–RNAi
	w^-;+; Apn–Gal4/UAS–AdipoR–Ri
	w^-; UAS–AdipoR–TRIP/+; Apn–Gal4/+
Fig. S1h:	w^-/Y;+; Apn–Gal4/UAS–GFP–RNAi
	w^-/Y; UAS–AdipoR–TRIP/+;UAS–GFP–RNAi/+
	w^-/Y; UAS–AdipoR–TRIP/+; Apn–Gal4/+
Fig. S1i:	w^-/Y; dilp2–Gal4/+; +/UAS–GFP–RNAi
	w^-/Y; dilp2–Gal4/+; +/UAS–AdipoR–Ri
Fig. S1k:	w^-/Y;+; Apn–Gal4/UAS–GFP–RNAi
	w^-/Y; UAS–hAdipoQ/+; UAS–GFP–RNAi/+
	w^-/Y; UAS–hAdipoQ/+; Apn–Gal4/+
Fig. S1l:	lpp–Gal4/Y; +;UAS–GFP–RNAi/+
	w^-/Y;+; UAS–AdipoR–Ri/UAS–GFP–RNAi
	lpp–Gal4/Y;+; UAS–AdipoR–Ri/+
	w^-/Y;+;MHC–Gal4/UAS–GFP–RNAi
	w^-/Y;+;MHC–Gal4/UAS–AdipoR–Ri
Fig. S1m–n:	w^-; +; +
	w^-; +;AdipoR–G6641
Fig. S2c:	lpp–Gal4/+/Y; +;UAS–grp78–RNAi/+
Fig. S2d:	lpp–Gal4/+/Y; +;UAS–sGFP–RNAi/+
Fig. S2e–e':	w^-;+; UAS–GFP–RNAi
Fig. S2f:	lpp–Gal4/+/Y; +;UAS–xbp1–GFP/+
Fig. S3a:	w^-;+; Apn–Gal4/UAS–GFP–RNAi
	w^-;+; UAS–AdipoR–Ri/UAS–GFP–RNAi
	w^-;+; Apn–Gal4/UAS–AdipoR–Ri
Fig. S3b:	yw–; Akh–Gal4/UAS–mCD8–GFP; +
Fig. S3c,d,f, g,j:	w^-;+; Apn–Gal4/UAS–GFP–RNAi
	w^-;+; Apn–Gal4/UAS–AdipoR–Ri
Fig. S3e,k:	w^-;+; Apn–Gal4/UAS–GFP–RNAi
	w^-;+; UAS–AdipoR–Ri/UAS–GFP–RNAi

w^{-};+; Apn-Gal4/UAS-AdipoR-Ri
Fig. S3h,i: w^{-};+; UAS-GFP-RNAi
Fig. S4a: w^{-};+; UAS-GFP-RNAi
Fig. S4b: w^{-};+; Apn-Gal4/UAS-GFP-RNAi
w^{-};+; Apn-Gal4/UAS-AdipoR-Ri
Fig. S4c: w^{-};tGPH/+; UAS-GFP-RNAi/+
Fig. S4d: w^{-};+; Apn-Gal4/UAS-GFP-RNAi
w^{-};+; UAS-AdipoR-Ri/UAS-GFP-RNAi
w^{-};+; Apn-Gal4/UAS-AdipoR-Ri
Fig. S4e: yw^{-}; UAS-Dcr2/+; gd2HF, Dilp2-Gal4/UAS-GFP-RNAi
yw^{-}; UAS-Dcr2/+; gd2HF, Dilp2-Gal4/UAS-AdipoR-Ri
Fig. S4f:w^{-} ; gd2HF/+; UAS-GFP-RNAi
w^{-} ; gd2HF/+; UAS-AdipoR-Ri
w^{-}; gd2HF/UAS-AdipoR-TRIP ; +

Table S1
"n" significance and values corresponding to Figures 1-4and Figures S1-S4.

Figure Ref.	n represents:	Values represented on Figures
1b	Total number of wing discs measured in 5 independent experiments	52 (Apn>), 45 (Apn>AdipoR-Ri)
1c	Total number of adult wings measured in 3 independent experiments	47 (Apn>), 51 (Apn>AdipoR-Ri)
1d	Number of groups of males weighed (collection of animals over several days, all weighed the same day)	Groups of 5 males: 20 (Apn>), 7 (>AdipoR-Ri), 9 (Apn>AdipoR-Ri), 10 (>AdipoR-act), 14 (Apn>AdipoR-act);<br>Groups of 10 males: 10 (lpp>), 5 (>hAdipoQand lpp>hAdipoQ)
1e-g	Number of independent measurements, each corresponding to the averaged intensity measured in 2-3neurons per APN cluster.	Apn>CalexA:16 (LSD and HSD); 17 (ND)<br>LSD: 12 (Apn>), 17 (Apn>AdipoR-Ri)<br>Apn>CalexA:18 (HSD), 20 (HSD +hAdipoQ in the food)
2a	Number of independent experiments	3
2b	Number of independent experiments	2
2c	Number of independent experiments	3
2d'	Number of measurements, each corresponding to the averaged intensity measured in 3-4neurons per APN cluster (2 independent ex-	25 (lpp>in LSD), 37 (lpp>in HSD), 29 (lpp>grp78-Riin HSD)

	vivo experiments at 2 different days)	
3a	Number of CNS/ring glands stained	18 (5 ind. exp.)
3b	Number of independent experiments	3
3c	Number of independent experiments	2
3d	Total number of wing discs measured in 3 independent experiments	21 (control in ND), 22 (control in ND +2ppm JHa)
3e	Number of groups of 5 males weighed (collection of animals over several days, all weighted the same day)	7 (control in ND), 8 (control in ND +2ppm JHa)
3f	Number of independent experiments realized to calculate the % of female survival rate	4<br><br>(Total of 1147 L1 larvae met27,gce25k/FM7;; Apn>x >GFP-Ricontrol cross and<br>Total of 1158 L1 larvae met27,gce25k/FM7;; Apn>x >AdipoR-Ri-Ri test cross)
3g	Number of groups of 15 females weighed (collection of animals over several days, all weighted the same day)	3
4a	Number of fat bodies explants	Minimum 8 (repeated 2 ind. exp.)
4a',b	Number of averaged independent measurements of GFP intensity in different pieces of fat tissue (See methods)	36-44(Apn>), 44 (Apn>AdipoR-Ri)<br>23-47(control), 23-37(control pretreated with JHa)
4c	Number of independent biological replicates measured in 3-4experiments (different days)	16 (elav>), 5 (elav>AdipoR-Ri), 10 (elav>AdipoR-act)<br>32 (Apn>), 21 (Apn>AdipoR-Ri), 17 (Apn>AdipoR-TRIP), 9 (Apn>AdipoR-act)
4d	Number of independent biological replicates measured in 2 experiments (different days)	12 (lpp>), 12 (lpp>hAdipoQ)
4e	Number of independent biological replicates measured in 3 experiments (different days)	18 (wo/JHa), 18 (W/JHa)
4f	Number of independent experiments	3
4g	Number of independent experiments	2

4h,i	Number of independent biological replicates measured in 3 independent experiments (different days)	24 (lpp>), 19 (lpp>grp78-Ri)<br>11 (lpp>), 9 (lpp>grp78)
S1a	Number of CNS/ring glands stained	18 (5 ind. exp.)
S1b	Number of CNS/ring glands stained	10 (2 ind. exp.)
S1c,c'	Number of measurements corresponding to the intensity measured in APN neurons. (2independent experiments)	24 (Apn>), 19 (Apn>AdipoR-Ri)
S1d,e	Number of independent experiments	2
S1f	Number of animals measured at each developmental time point	72hrs AEL: 23 (Apn>), 27 (>AdipoR-TRIP), 21 (>AdipoR-Ri), 28 (Apn>AdipoR-TRIP), 26 (Apn>AdipoR-Ri);<br><br>96hrs AEL: 55 (Apn>), 35 (>AdipoR-TRIP), 24 (>AdipoR-Ri), 35 (Apn>AdipoR-TRIP), 27 (Apn>AdipoR-Ri);<br><br>120hrs AEL: 82 (Apn>), 46 (>AdipoR-TRIP), 40 (>AdipoR-Ri), 99 (Apn>AdipoR-TRIP), 67 (Apn>AdipoR-Ri);
S1g	Number of adult wings measured<br>(3 independent experiments)	47 (Apn>), 51 (Apn>AdipoR-TRIP)
S1h,i	Number of groups of 10 males weighed (collection of animals over several days, all weighed the same day)	7 (Apn>), 3 (>AdipoR-TRIP), 10 (Apn>AdipoR-TRIP)<br>9 (dilp2>), 7 (dilp2>AdipoR-Ri)
S1j	Number of independent experiments	5
S1k	Number of groups of 5 males weighed (collection of animals over several days, all weighed the same day)	23 (Apn>), 14 (>hAdipoQ), 20 (Apn>hAdipoQ)
S1l	Number of groups of 15 males weighed (collection of animals over several days, all weighed the same day)	4 (>AdipoR-Ri), 4 (lpp>), 2 (lpp>AdipoR-Ri), 7 (mhc>), 3 (mhc>AdipoR-Ri)
S1m	Number of animals measured at each developmental time point	72hrs AEL: 17 (control), 15 ($AdipoR^{M}$)<br>96hrs AEL: 10 (control), 8 ($AdipoR^{M}$)<br>120hrs AEL: 16 (control), 18 ($AdipoR^{M}$)
S1n	Number of independent experiments	4

S2d,e	Number of independent experiments	3
S2f,f'	Number of independent experiments	2 (control versus DTT)<br>3 (LSD, HSD)
S2g,h	Number of independent experiments	2
S3a	Number of independent experiments	2
S3b	Number of CNS/ring glands stained	12 (in 2 ind. exp.)
S3c	Number of CNS/ring glands stained	13 (Apn>), 12 (Apn>AdipoR-Ri)<br>(2 ind. exp.)
S3d	Number of independent experiments	2
S3f-h	Number of independent experiments (different days)	3-4
S3i	Number of CNS/ring glands stained	10
S3j	Number of independent experiments	2
S3k	Number of indeoendent replicates	2-3
S4a	Number of independent experiments	2
S4b	Number of averaged independent measurements of GFP intensity in different pieces of fat tissue	13 (Apn>), 14 (Apn>AdipoR-Ri)<br>(minimum 8 fat pieces and repeated in 2 ind. exp.)
S4c	Number of fat bodies explants	Minimum 8 (repeated 2 ind. exp.)
S4d	Number of independent experiments	2
S4e,f	Number of independent biological replicates measured in 2-3experiments (different days)	3 exp.: 18 (dilp2>, dilp2>AdipoR-Ri)<br>2 exp.: 4 (control), 9 (>AdipoR-Ri), 8 (>AdipoR-TRIP)

Table S2
Primers names and sequences

Primers name (qRT-PCR)	Sequences	Supplier
rp49	Forward CTTCATCCGCCACCAGTC<br>Reverse CGACGCACTCTGTTGTCG	Sigma Aldrich
grp78	Forward CGAGGAGGAGGAAAAGGA<br>Reverse CGTCCGTTCTTGTACACACC	Sigma Aldrich
sugarbabe	Forward CGTGATCATGAAGGCTCGT<br>Reverse CTGCTGCTGCAGGACTTG	Sigma Aldrich
jhi-26	Forward ATACGACGGGCAAAAGTTTC<br>Reverse GGTGGCATAGCAGGTGTTTT	Sigma Aldrich
kr-h1	Forward TCACACATCAAGAAGCAACT<br>Reverse GCTGGTTGGCGCAATAGTAA	Sigma Aldrich

Inr	Forward TGTCAGCTGCACAATAATAGGC<br>Reverse TGCACTTTTCAGGGCATTT	Sigma Aldrich
AdipoR	Forward TGCCTAGGATTCTCATTCGCTTTTCACACA<br>Reverse GCTGGCGTATTTATGAGCTTTGGCCTGTCG	Sigma Aldrich
Xbp1s	Forward ACCAACCTTGGATCTGC<br>Reverse CGCCAAGCATGTCTTGT	Eurofins Genomics
Xbp1t	Forward TGGGAGGAGAAAGTGCA<br>Reverse TCCGTTCTGTCTGTCAG	Eurofins Genomics
Akh	Forward AGCCGTGCTCTTCATGCT<br>Reverse AAAGGTTCCAGGACCAGCTC	Sigma Aldrich
E78	Forward CATGTGGCCCGGTTGATC<br>Reverse CGTTGACAAAGTCAGAATCGTAGAG	Sigma Aldrich
ptth	Forward CGTAATGGATGCTGCTGCT<br>Reverse GGCATCATGCTCGATCTGT	Sigma Aldrich
dilp2	Forward ATCTGGACGCCCTCAATCC<br>Reverse TCCCAGGAAAGAGGGCACTT	Sigma Aldrich
Primers name (Gateway cloning)	Sequences	Supplier
UAS-hAdipoQ-Flag	Forward CACCATGGTGTTGCTGGGAGCTGTTCTACTGCTA<br>Reverse GTTGGTGTCATGGTAGAGAAGAAAGCCTGT	Sigma Aldrich
UAS-AdipoR-Myc	Forward CACCATGCAGACCCAGCCGGAGGTTATAGTGCGT<br>Reverse AAAGGTTATTGGTTCGATGGGAACAGTGC	Sigma Aldrich
UAS-grp78-HA	Forward CACCTTCACTTTGAAGCGTCGAGAAAGCACATTA<br>Reverse GTTGTTGTTGTTGTTGTGGCTACTGCGTT	Sigma Aldrich

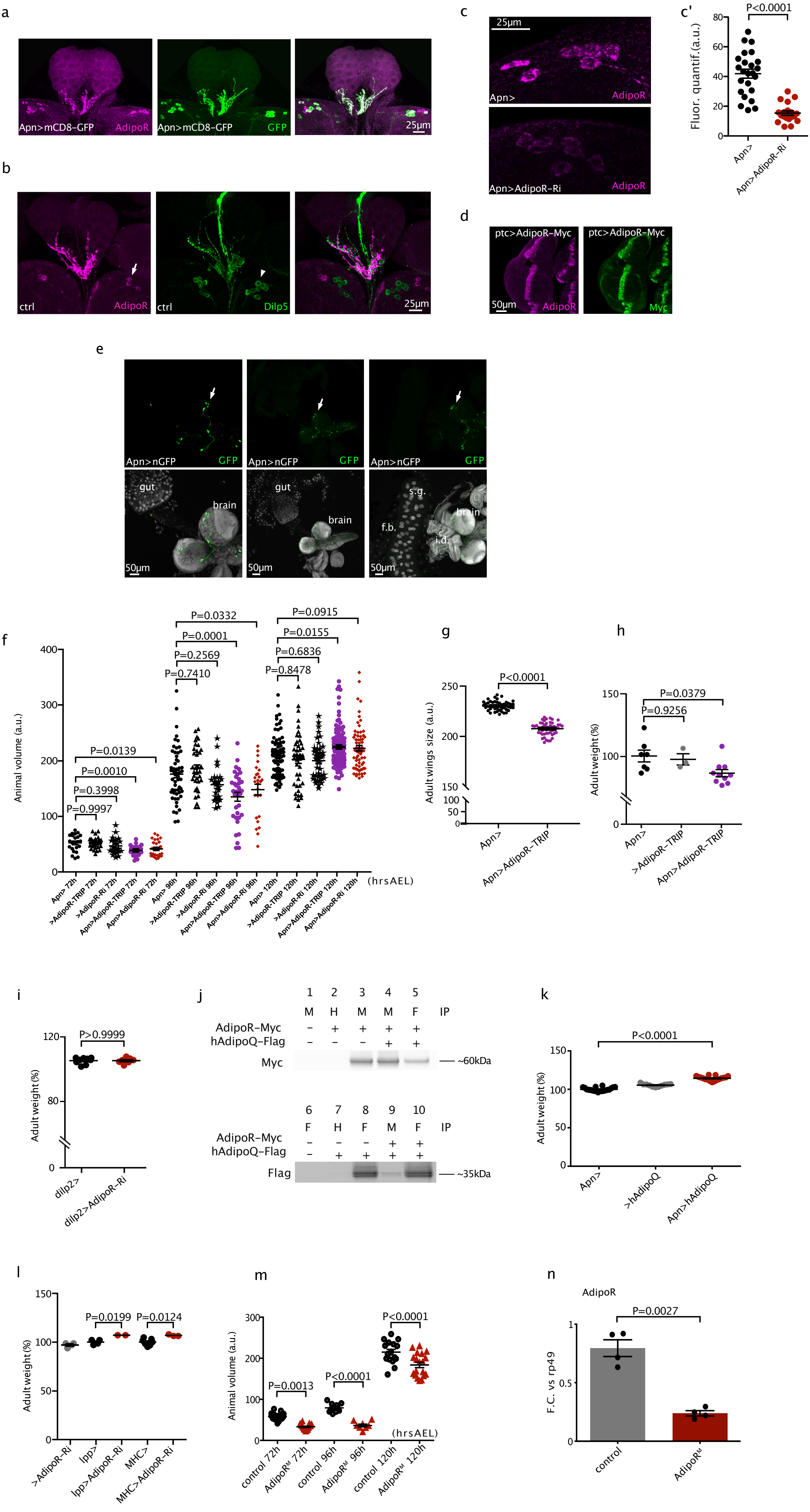
a
Apn>mCD8-GFP
AdipoR
Apn>mCD8-GFP
GFP
25μm
b
ctrl
AdipoR
ctrl
Dilp5
25μm
c
25μm
Apn>
AdipoR
Apn>AdipoR-Ri
AdipoR
c'
P<0.0001
Fluor. quantif.(a.u.)
Apn>
Apn>AdipoR-Ri
d
ptc>AdipoR-Myc
ptc>AdipoR-Myc
50μm
AdipoR
Myc
e
Apn>nGFP
GFP
gut
brain
s.g.
f.b.
i.d.
50μm
f
Animal volume (a.u.)
P=0.0139
P=0.0010
P=0.3998
P=0.9997
P=0.0332
P=0.0001
P=0.2569
P=0.7410
P=0.0915
P=0.0155
P=0.6836
P=0.8478
Apn> 72h
>AdipoR-TRIP 72h
>AdipoR-Ri 72h
Apn>AdipoR-TRIP 72h
Apn>AdipoR-Ri 72h
Apn> 96h
>AdipoR-TRIP 96h
>AdipoR-Ri 96h
Apn>AdipoR-TRIP 96h
Apn>AdipoR-Ri 96h
Apn> 120h
>AdipoR-TRIP 120h
>AdipoR-Ri 120h
Apn>AdipoR-TRIP 120h
Apn>AdipoR-Ri 120h
(hrsAEL)
g
P<0.0001
Adult wings size (a.u.)
Apn>
Apn>AdipoR-TRIP
h
P=0.0379
P=0.9256
Adult weight (%)
Apn>
>AdipoR-TRIP
Apn>AdipoR-TRIP
i
P>0.9999
Adult weight (%)
dilp2>
dilp2>AdipoR-Ri
j
IP
AdipoR-Myc
hAdipoQ-Flag
Myc
~60kDa
Flag
~35kDa
k
P<0.0001
Adult weight (%)
Apn>
>hAdipoQ
Apn>hAdipoQ
l
P=0.0199
P=0.0124
Adult weight (%)
>AdipoR-Ri
lpp>
lpp>AdipoR-Ri
MHC>
MHC>AdipoR-Ri
m
Animal volume (a.u.)
P=0.0013
P<0.0001
P<0.0001
(hrsAEL)
control 72h
AdipoR^M 72h
control 96h
AdipoR^M 96h
control 120h
AdipoR^M 120h
n
AdipoR
P=0.0027
F.C. vs rp49
control
AdipoR^M

Fig. S1: **a**, Double immunostaining anti-AdipoR (magenta) and anti-GFP (green) on Apn>mCD8-GFP larval brains and ring glands. (n=18in five independent experiments). **b,** Double immunostaininganti-AdipoR (Apn, magenta, arrow) and anti-Dilp5 (IPCs, green, arrowhead). n=2 independent immunostaining experiments. **c**, Immunostaining anti-AdipoR on larval brains showing strong reduction in Apn>AdipoR-Ricompared to control brains (Apn>). (minimum n=10in two independent immunostaining experiments). **c'**, Quantification of AdipoR staining (related to **c**) in Apn> (n=24), Apn>AdipoR-Ri(n=19).n=numberof independent measurements. Statistical significance was tested using two-sided Mann-Whitney test. **d**, Double immunostaining anti-AdipoR (magenta) and anti-Myc (green) on ptc>AdipoR-Myclarval imaginal discs. n=2independent immunostaining experiments. **e**, GFP staining in larval tissues showing Apn>nGFP(nuclear-GFP) expression in the larval brain (arrow, upper panels). No staining is observed in the gut, the salivary glands (s.g.), the fat body (f.b.) or the imaginal discs (i.d.) (lower panels). Tissues are labelled with anti-GFP and DAPI. n=2independent immunostaining experiments. **f**, Larval size is reduced at 72hand 96hAEL in Apn>AdipoR-Ri(72hn=27; 96h n=27, red squares), and Apn>AdipoR-TRIP (72h n=26; 96h n=35, purple points) compared to controls Apn> (72h n=23; 96h n=55, black points), >AdipoR-Ri(72h n=28; 96h n=24, black triangles) and >AdipoR-TRIP(72h n=21; 96h n=35, black stars). Pupal volume (120h AEL) is slightly reduced in Apn>AdipoR-TRIP(n=99, purple points) but not in Apn>AdipoR-Ri(n=67, red squares), compared to controls (Apn> n=82, black points; >AdipoR-Ri n=40, black triangles and >AdipoR-TRIP n=46, black stars). n=numberof independent animals. Statistical significance was tested using ordinary one-way ANOVA with Dunnett's multiple comparisons test. **g**, Wing size reduction in Apn>AdipoR-RiTRIP(n=51, purple) compared to Apn> (n=47, black). n=number of biologically independent samples from 3 independent experiments. Statistical significance was tested using ordinary two-way ANOVA with Dunnett's multiple comparisons test. **h**, 13% reduction of adult weight in Apn>AdipoR-Ri-TRIP(n=10, purple) compared to controls (Apn> n=3, black; >AdipoR-TRIPn=3, grey). n=numberof groups of 10 males. Statistical significance was tested using ordinary one-way ANOVA with Tukey's multiple comparisons test. **i,** No difference in adult weight between dilp2> (n=9, black) and dilp2>AdipoR-Ri (n=7, red). n=number of groups of 10 males. Statistical significance was tested using two-sided unpaired t-test with Welch's correction. **j**, Co-immunoprecipitations (co-IP) of AdipoR-Myc, and hAdipoQ-Flagexpressedin S2 cells. Myc (M) and Flag (F) IPs are used to visualize AdipoR-Myc (1-5)or hAdipoQ-Flag (6-10). n=5independent experiments. **k**, 14% increase in adult weight in Apn>hAdipoQ (n=20, red) compared to controls (Apn>n=23, black and >hAdipoQn=14, grey). n=numberof groups of 5 males. Statistical significance was tested using ordinary one-way ANOVA with Tukey's multiple comparisons test. **l**, Size increase induced by reduction of AdipoR in the fat (lpp>AdipoR-Rin=2, red; lpp>n=4, black) or in the muscles (MHC>AdipoR-Ri n=3, red; MHC>n=7, black). n=number of groups

of 15 males measured. Statistical significance was tested using ordinary one-way ANOVA with Tukey's multiple comparisons test. **m**, Size reduction in AdipoR^M larvae (72h AEL, n=15; 96h AEL, n=8, red triangles) and pupae (120h AEL, n=18; red triangles) compared to w controls (72h AEL, n=17; 96h AEL, n=10; 120h AEL, n=16; black points). n=number of independent animals. Statistical significance was tested using ordinary one-way ANOVA with Bonferroni's multiple comparisons test. **n**, AdipoR expression on whole larvae in AdipoR^M mutants (red) compared to control (black). qRT-PCR profiles, Fold Changes (F.C.) normalized to rp49. n=4 independent experiments. Statistical significance was tested using two-sided unpaired t-test with Welch's correction. a.u.= arbitrary units. Data are presented as mean values +/_SEM. P values are indicated in the panels. Source data are provided as a Source Data file.

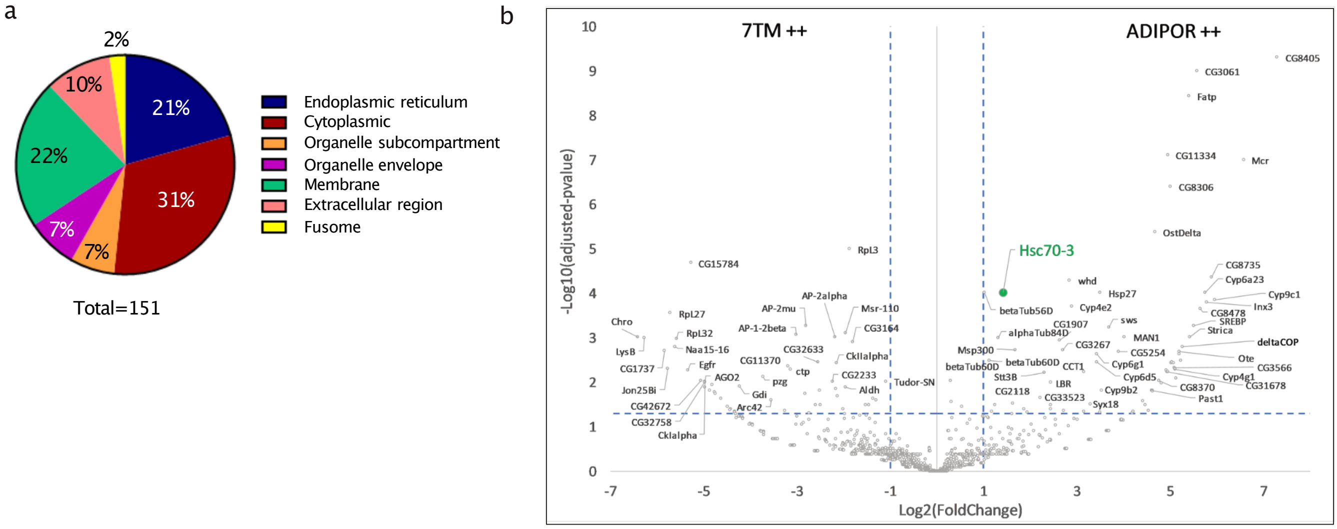

c

gene	pupal lethality w/ *ppl>*
Mcr	++
CG30159	-
Manf	+
Ost48	++
Fatp	-
OstDelta	++
Stt3B	-
CG8370	+
Hsc70-3	++
CG7601	-
CG3061	-
CG8478	-
CG11334	-
CG8735	-
CG44245	-
CG33523	++
Strica	-
CG7461	-
Srebp	-
kkv	-
Msp300	-
CG8405	-
sws	-
Mpcp1	-
Ote	-
Syx18	+

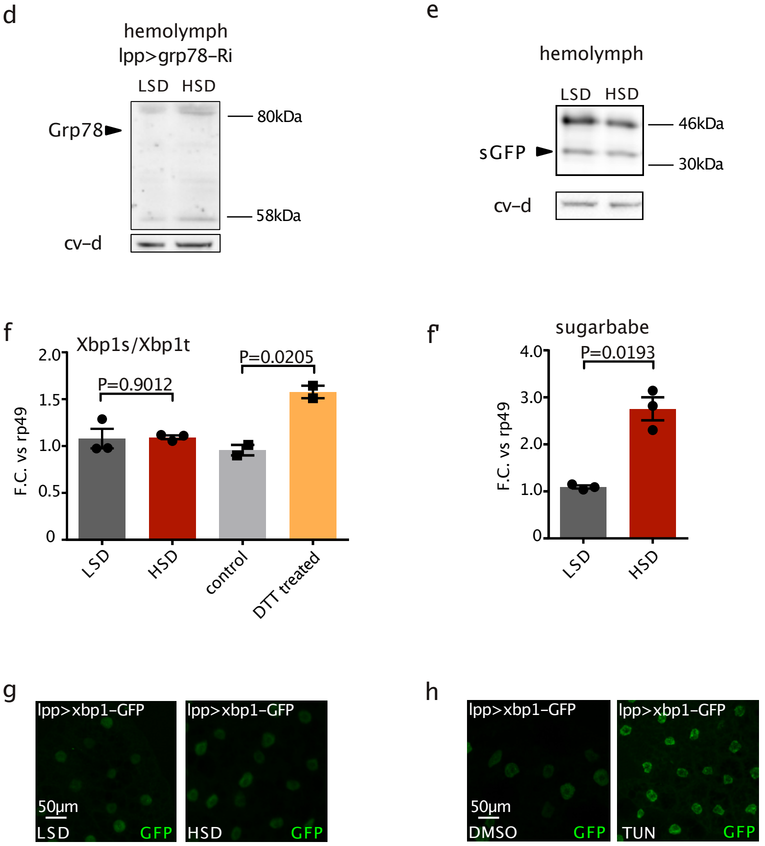

Fig.S2: **a**, Pie chart illustrating the distribution in % of the 151hits identified in the massspectrometry by subcellular localization. **b**, Volcano plot showing the distribution of the Grp78 (Hsc70-3in green) and the other hits according to their enrichment value between the control and the test condition. Fold change and adjusted p-valueswere calculated with a two-sidedWald test with Benjamini and Hochberg correction for multi-testing. For each hit, adjusted p-value (FDR, -Log10(FDR))and Log2fold change (FC) are shown. Grp78 (Hsc70-3): 1,43 Log2(FoldChange) with P value=$9.65.10^{-05}$ and -Log10(FDR)=4,02.**c**, Table recapitulating the pupal lethality quantified by reducing expression of the selected candidate genes in the larval fat body (lpp>gene-Ri). **d**, No Grp78 is detected in the hemolymph of low (LSD) and high (HSD) sugar diet-fed lpp>grp78-Rilarvae. Cv-d is a control for hemolymph proteins. **e**, secreted-GFP (sGFP) immunodetection in the hemolymph of low (LSD) and high (HSD) sugar diet-fed larvae. Cv-dis a control for hemolymph proteins. (experiment was repeated 3 times independently with similar resultsin **d,e**). **f**, Ratio of splicedXbp1over total Xbp1(Xbp1s/Xbp1t) in whole larvae fed LSD (dark grey) or HSD (red) and in LSD fed larvae treated (orange) or not (light grey) with 5mM DTT.**f'**, sugarbabe expressionas a control of HSD (related to **f**) (qRT-PCRprofiles, Fold Changes (F.C.) normalized to rp49). In (**f,f'**) n=2 (LSD/HSD) and 3 (DTT treatment) independent experiments. Statistical significance was tested using two-sided unpaired t-test with Welch's correction (**f,f'**). **g**, No change in the subcellular localization of the Xbp1-GFPin fat bodies from lpp>xbp1-GFPlarvaereared on LSD or HSD. **h**, Nuclear localization increase of Xbp1-GFPin fat bodies of lpp>xbp1-GFPlarvaereared on LSD and treated 1hr with tunicamycin (TUN) or DMSO as a control. **g** and **h**, anti-GFP immunostaining of fat bodies; n=2independent experiments.

Data are presented as mean values +/_SEM. P values are indicated in the panels. Source data are provided as a Source Data file.

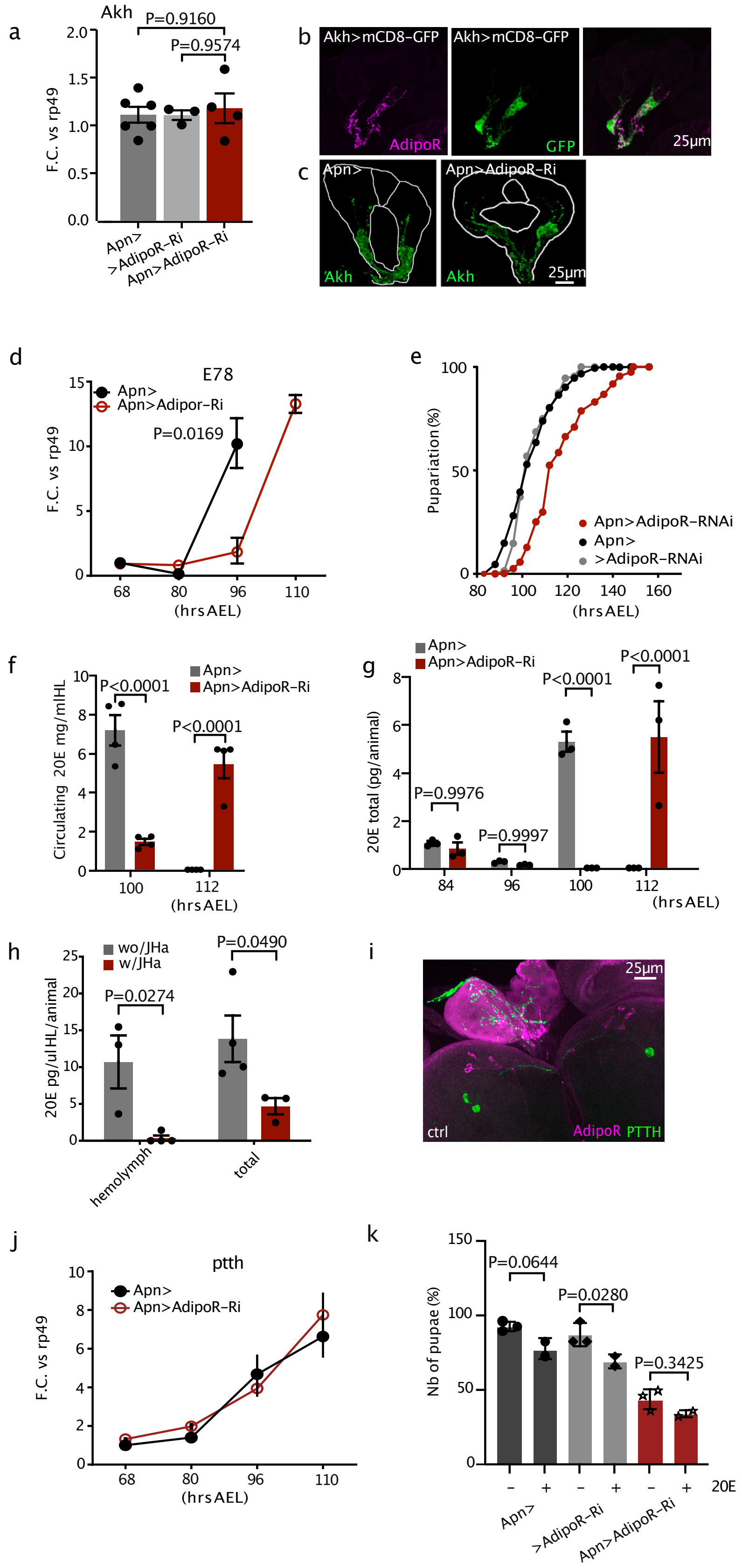
a
Akh
P=0.9160
P=0.9574
F.C. vs rp49
2.0
1.5
1.0
0.5
0.0
Apn>
>AdipoR-Ri
Apn>AdipoR-Ri
b
Akh>mCD8-GFP
Akh>mCD8-GFP
AdipoR
GFP
25µm
c
Apn>
Apn>AdipoR-Ri
Akh
Akh
25µm
d
E78
Apn>
Apn>Adipor-Ri
P=0.0169
F.C. vs rp49
15
10
5
0
68
80
96
110
(hrs AEL)
e
Pupariation (%)
100
50
0
80
100
120
140
160
(hrs AEL)
Apn>AdipoR-RNAi
Apn>
>AdipoR-RNAi
f
Circulating 20E mg/mlHL
Apn>
Apn>AdipoR-Ri
P<0.0001
P<0.0001
10
8
6
4
2
0
100
112
(hrs AEL)
g
Apn>
Apn>AdipoR-Ri
20E total (pg/animal)
P=0.9976
P=0.9997
P<0.0001
P<0.0001
8
6
4
2
0
84
96
100
112
(hrs AEL)
h
wo/JHa
w/JHa
20E pg/ul HL/animal
P=0.0274
P=0.0490
25
20
15
10
5
0
hemolymph
total
i
25µm
ctrl
AdipoR
PTTH
j
ptth
Apn>
Apn>AdipoR-Ri
F.C. vs rp49
10
8
6
4
2
0
68
80
96
110
(hrs AEL)
k
Nb of pupae (%)
P=0.0644
P=0.0280
P=0.3425
150
100
50
0
–
+
–
+
–
+
20E
Apn>
>AdipoR-Ri
Apn>AdipoR-Ri

Fig. S3: a, No change in Akh expression in Apn>AdipoR-Rilarvae (red) compared to controls (Apn>, black and >AdipoR-Ri,grey). Statistical significance was tested using ordinary two-way ANOVA with Sidak's multiple comparisons test. n=3-6biologicallyindependent replicates (in 2 ind. exp.). **b**, Double immunostaining anti-AdipoR (magenta) and anti-GFP (green) on Akh>mCD8-GFPlarval ring glands (n=12 in two ind. exp.). **c**, Immunostaining anti-Akh on larval ring glands showing no difference in Apn>AdipoR-Ri(n=13) compared to control brains (Apn> n=12). **d**, E78 expression on whole larvae Apn>AdipoR-Ri(red) and Apn> (black). qRT-PCR profiles, Fold Changes (F.C.) normalized to rp49. Statistical significance was tested using ordinary two-way ANOVA with Sidak's multiple comparisons test. n=3biologically independent replicates per time point (2 ind. exp. and 3 additional ind. exp. for the 96h AEL time point). **e**, Pupariation time at 29ºC in Apn>AdipoR-Ri(red) compared to Apn>(black) and >AdipoR-Ri(grey). Statistical significance was tested using ordinary two-way ANOVA with Tukey's multiple comparisons test (****p<0,0001 for each time point). **f,g,h**, 20E levels in hemolymph (HL) at 100h and 112h AEL (in **f**) and total levels at 84h, 96h, 100 and 112h AEL (in **g**) in Apn>(grey) and Apn>AdipoR-Ri(red) animals. n=4(**f**) and 3 (**g**) independent experiments. Hemolymph and total 20E levels in larvae fed (red) or not (grey) with JHa at 2ppm(**h**). n=3-4independentexperiments.Statistical significance was tested using ordinary two-way ANOVA with Sidak's multiple comparisons test (f-h). **i**, Double immunostaining anti-AdipoR (magenta) and anti-PTTH(green) on brains and ring glands (n=10). **j**, ptth expression on whole larvae Apn>AdipoR-Ri(red) and Apn> (black). qRT-PCR profiles, Fold Changes (F.C.) normalized to rp49. n=3biologically independent replicates per time point (2 ind. exp.). **k**, Pupal achievement in animals fed w/20E. Apn>(dark grey), >AdipoR-Ri(light grey) and Apn>AdipoR-Ri (red) and n=3independent measurements of pupal achievement in a vial in which 30 L1 were picked. Statistical significance was tested using ordinary one-way ANOVA with Bonferroni's multiple comparisons test. Data are presented as mean values +/_SEM. P values are indicated in all panels. Source data are provided as a Source Data file.

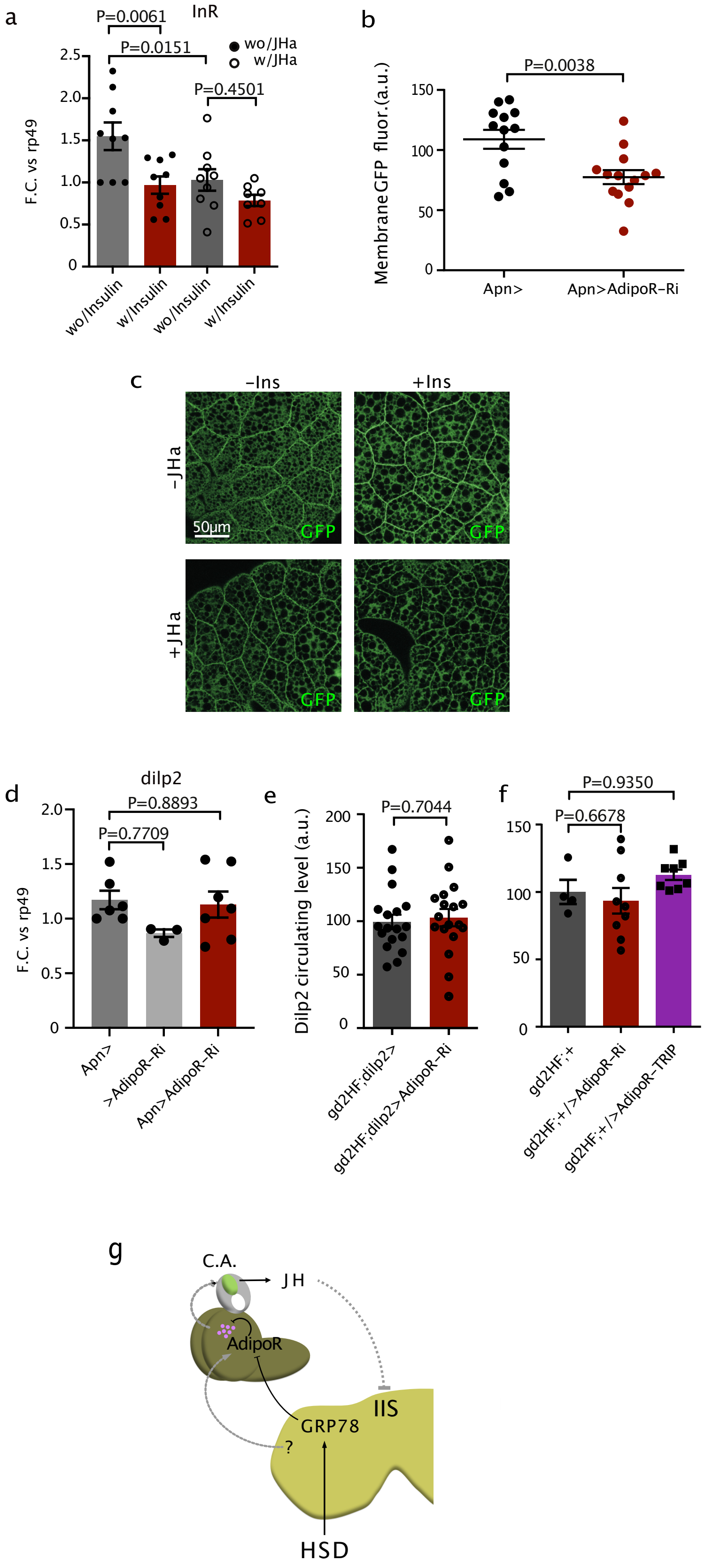
a
InR
P=0.0061
P=0.0151
P=0.4501
wo/JHa
w/JHa
F.C. vs rp49
2.5
2.0
1.5
1.0
0.5
0
wo/Insulin
w/Insulin
wo/Insulin
w/Insulin
b
P=0.0038
Membrane GFP fluor.(a.u.)
150
100
50
0
Apn>
Apn>AdipoR-Ri
c
-Ins
+Ins
-JHa
+JHa
50μm
GFP
GFP
GFP
GFP
d
dIlp2
P=0.8893
P=0.7709
F.C. vs rp49
2.0
1.5
1.0
0.5
0
Apn>
>AdipoR-Ri
Apn>AdipoR-Ri
e
P=0.7044
Dilp2 circulating level (a.u.)
200
150
100
50
0
gd2HF;dilp2>
gd2HF;dilp2>AdipoR-Ri
f
P=0.9350
P=0.6678
150
100
50
0
gd2HF;+
gd2HF;+/>AdipoR-Ri
gd2HF;+/>AdipoR-TRIP
g
C.A.
JH
AdipoR
IIS
GRP78
?
HSD

Fig. S4: a, InR expression in larval fat bodies pretreated (points) or not (circles) JHa and incubated -/+ human insulin. qRT-PCR profiles, Fold Changes (F.C.), related to Figure 4a. Statistical significance was tested using ordinary one-way ANOVA with Sidak's multiple comparisons test. Data are presented as mean values +/_SEM. **b**, GFP quantification of membrane GFP staining in tGPH fat bodies from Apn> (n=13, black) and Apn>AdipoR-Ri(n=14, red) larvae prior treatment (related to Figure 4a) in 2 independent experiments. Statistical significance was tested using two-sided unpaired t-test with Welch's correction. **c**, GFP localization at the membrane in tGPH fat body explants, as a marker for insulin response. Pretreatment -/+JHa and incubation -/+human insulin, related to Figure 4b. Fat bodies are stained with anti-GFP. n=2 independent immunostaining experiments. **d**, dilp2 expression on dissected larval brains in Apn>AdipoR-Ri(red) compared to Apn>(black) and >AdipoR-Ri(grey). qRT-PCR profiles, Fold Changes (F.C.) normalized to rp49. n=3 biologically independent replicates (in 2 ind. exp.). Statistical significance was tested using ordinary one-way ANOVA with Dunnett's multiple comparaisons test. **e-f**, Measure of hemolymph Dilp2 by ELISA in gd2HF;dilp2>AdipoR-Ri(n=18, red) compared to gd2HF;dilp2>(n=18, grey) (in three ind. exp.; **e**) and in control RNAi conditions gd2HF;+ (n=4, grey), gd2HF;+>AdipoR-Ri(n=9, red) and gd2HF;+>AdipoR-TRIP(n=8, purple) (in two ind. exp.; **f**). n=number of independent measurements. Statistical significance was tested using ordinary two-way ANOVA with Sidak's multiple comparisons test. **g,** Proposed working model: in high sugar diet (HSD), secreted Grp78 from the fat body interacts with AdipoR and its unknown agonist in the APNs. This counteracts AdipoR agonist action and releases the inhibition of AdipoR on APNs. As a consequence, peripheral JH signaling increase, possibly through the release of JH from the CA. This in turn decreases peripheral insulin/IGF signaling (IIS). Data are presented as mean values +/_SEM. P values are indicated in all panels. a.u.=arbitrary units. Source data are provided as a Source Data file.
